# Interspinous Spacer versus Traditional Decompressive Surgery for Lumbar Spinal Stenosis: A Systematic Review and Meta-Analysis

**DOI:** 10.1371/journal.pone.0097142

**Published:** 2014-05-08

**Authors:** Ai-Min Wu, Yong Zhou, Qing-Long Li, Xin-Lei Wu, Yong-Long Jin, Peng Luo, Yong-Long Chi, Xiang-Yang Wang

**Affiliations:** 1 The Department of Spinal Surgery, Second Affiliated Hospital of Wenzhou Medical University, Zhejiang Spinal Research Center, Wenzhou, Zhejiang, People's Republic of China; 2 The First Medical College, Wenzhou Medical University, Wenzhou, Zhejiang, People's Republic of China; 3 Institute of Digitized Medicine, Wenzhou Medical University, Wenzhou, Zhejiang, People's Republic of China; University of Toronto, Canada

## Abstract

**Background:**

Dynamic interspinous spacers, such as X-stop, Coflex, DIAM, and Aperius, are widely used for the treatment of lumbar spinal stenosis. However, controversy remains as to whether dynamic interspinous spacer use is superior to traditional decompressive surgery.

**Methods:**

Medline, Embase, Cochrane Library, and the Cochrane Controlled Trials Register were searched during August 2013. A track search was performed on February 27, 2014. Study was included in this review if it was: (1) a randomized controlled trial (RCT) or non-randomized prospective comparison study, (2) comparing the clinical outcomes for interspinous spacer use versus traditional decompressive surgery, (3) in a minimum of 30 patients, (4) with a follow-up duration of at least 12 months.

**Results:**

Two RCTs and three non-randomized prospective studies were included, with 204 patients in the interspinous spacer (IS) group and 217 patients in the traditional decompressive surgery (TDS) group. Pooled analysis showed no significant difference between the IS and TDS groups for low back pain (WMD: 1.2; 95% CI: −10.12, 12.53; P = 0.03; I^2^ = 66%), leg pain (WMD: 7.12; 95% CI: −3.88, 18.12; P = 0.02; I^2^ = 70%), ODI (WMD: 6.88; 95% CI: −14.92, 28.68; P = 0.03; I^2^ = 79%), RDQ (WMD: −1.30, 95% CI: −3.07, 0.47; P = 0.00; I^2^ = 0%), or complications (RR: 1.39; 95% CI: 0.61, 3.14; P = 0.23; I^2^ = 28%). The TDS group had a significantly lower incidence of reoperation (RR: 3.34; 95% CI: 1.77, 6.31; P = 0.60; I^2^ = 0%).

**Conclusion:**

Although patients may obtain some benefits from interspinous spacers implanted through a minimally invasive technique, interspinous spacer use is associated with a higher incidence of reoperation and higher cost. The indications, risks, and benefits of using an interspinous process device should be carefully considered before surgery.

## Introduction

Degenerative lumbar spinal stenosis is common in the elderly population, and many affected individuals have pain and neurogenic intermittent claudication. Decompressive surgery is recommended for their treatment [1], [2]. Flexion tends to relieve symptoms for some patients. Therefore, dynamic devices have been designed to limit spinal extension. These devices include interspinous spacers, such as the X-stop, Coflex, DIAM, and Aperius devices [3], [4], [5].

However, controversy remains about whether interspinous spacers produce better or worse outcomes than traditional decompressive surgery [6], [7]. Richards et al. reported that implanted interspinous spacers could increase the spinal canal area, as well as the width and area of the intervertebral foramen [8]. Zucherman et al. [9] reported good functional improvement and pain relief after the implantation of interspinous spacers. However, Bowers et al. [10] noted that the procedure carried a high rate of complications, which was separately reported by Kim et al. [11].

The aim of this study was to compare the clinical outcomes of interspinous spacer use to traditional decompressive surgery.

## Methods

### Search strategy

Electronic databases of Medline, Embase, Cochrane Library, and the Cochrane Controlled Trials Register were searched without restriction for publication date or language during August 2013. The following keywords were used, in combination with Boolean operators: “lumbar spinal stenosis,” “neurogenic intermittent claudication,” “interspinous spacer,” “X-stop,” “Coflex,” “DIAM,” “Wallis,” “Aperius,” and “decompressive surgery”. Related articles and reference lists were searched to avoid omissions. A track search was performed on February 27, 2014, to add any new publications.

### Eligibility criteria

A study was included in the analysis if it was: (1) a randomized controlled trial (RCT) or a non-randomized prospective comparative study, (2) comparing the clinical outcomes of interspinous spacer use versus traditional decompressive surgery, (3) in at least 30 patients, (4) with a follow-up period of at least 12 months. Two authors (AMW and YZ) assessed the potentially eligible studies independently. Any disagreement was discussed and resolved with a third independent author (LQL).

### Data extraction

Data were independently extracted by two investigators (XLW and YLJ) using a standardized form (**Table S1**). Collected data included the publication date, study design, sample size, follow-up duration, interventions, complications, incidence of reoperation, and clinical outcomes, including low back pain, leg pain, the Oswestry disability index (ODI), and the Roland disability questionnaire (RDQ).

### Risk of bias assessment

Risk of bias was assessed with the Downs and Black checklist [12]. The quality levels of randomized and non-randomized studies of healthcare interventions were assessed with 27 questions, as shown in **Table S2**.

### Statistical analysis

Meta-analyses were performed in the RevMan 5.2 software (Cochrane Collaboration, Software Update, Oxford, UK), according to the recommendations of the Cochrane Collaboration. Another author independently checked the data before the analysis was performed. Risk ratios (RRs) were calculated for binary outcomes and weighted mean differences (WMDs) for continuous outcomes, along with the 95% confidence intervals (CIs). Heterogeneity was evaluated by chi-squared and I^2^ tests. Acceptable heterogeneity was defined by a P-value of <0.01 for the chi-squared test and <30% for the I^2^ test. A sensitivity analysis was performed, in which the possible effects of removing one study from the analysis were evaluated. Homogeneous data were pooled with a fixed-effects model. Heterogeneous data were assessed by a random-effects model.

## Results

### Studies included and risk of bias

**Figure 1:**
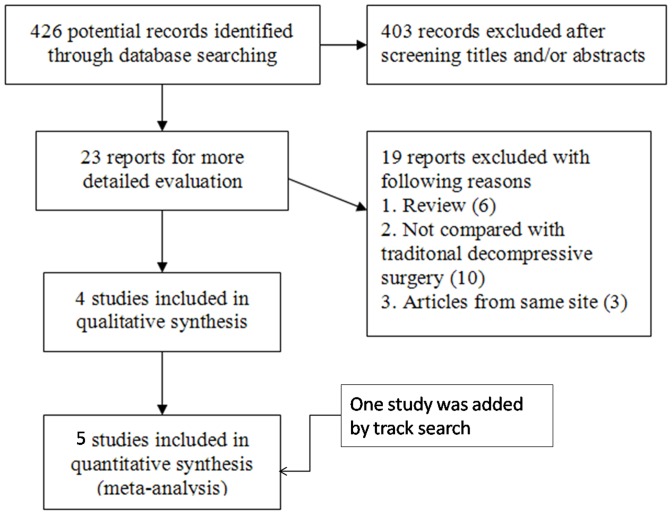
Flowchart of the study selection process.

The first search strategy identified 426 potential studies, of which 422 reports were excluded. One RCT [13] and three non-randomized prospective studies [14], [15], [16] were included according to the eligibility criteria. Another RCT was included by the track search [17] (**Fig. 1**). In total, there were 208 patients in the interspinous spacer (IS) group and 217 patients in the traditional decompressive surgery (TDS) group. The characteristics of all five included studies are shown in **Table 1**. The risk of bias assessment according to the Downs and Black checklist of all included studies is shown in **Table S2**.

**Table 1:** Characteristics of the four included studies.

Characteristic	Stromqvist 2013	Beyer 2013	Richter 2012	Kim 2007	Moojen 2013
Study design	RCT	Non-RCT	Non-RCT	Non-RCT	RCT
Follow-up duration	24 months	24 months	24 months	12 months	12 months
Participants	100 patients Age: 69 (49–89) years	45 patients Age: 69.3±9.7 years	62 patients Age: 68 (52–79) years	62 patients Age: 50 (20–81) years	159 patients Age: 63 (45–83) years
Intervention	IS = 50 TDS = 50 IS (X-Stop)	IS = 12 TDS = 33 IS (Aperius)	IS = 31 TDS = 31 IS (Coflex)	IS = 31 TDS = 31 IS (DIAM)	IS = 80 TDS = 79 IS (distraXion)
Outcomes	VAS of low back pain and leg pain, complications and reoperation	VAS of low back pain and leg pain, ODI and complications	ODI, RDQ, complications and reoperation	VAS of low back pain and leg pain, complications	VAS of low back pain and leg pain, RDQ, complications, reoperation

**Note:** RCT: Randomized controlled trial; IS: Interspinous spacer group; TDS: Traditional decompressive surgery group; VAS: Visual analogue scale; ODI: Oswestry disability index; RDQ: Roland disability questionnaire.

### Clinical outcomes

**Figure 2:**
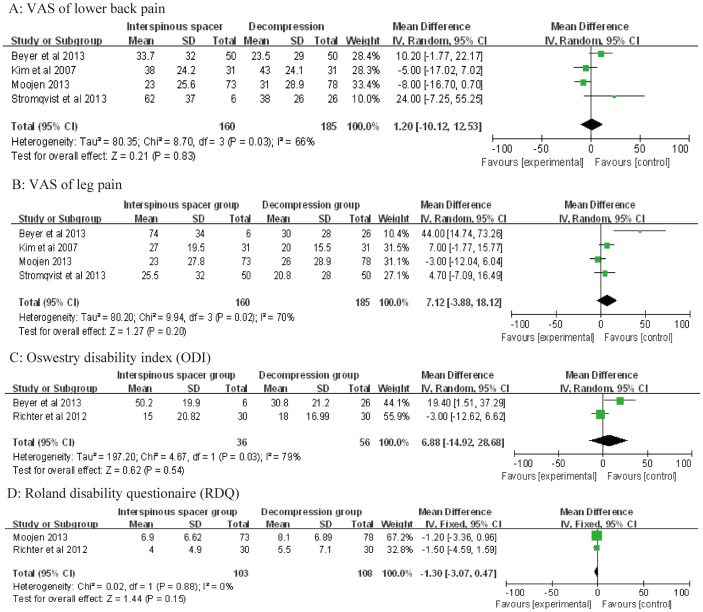
Forest plot showing the meta-analysis of visual analogue scale (VAS) scores for low back pain (A) and leg pain (B), the Oswestry disability index (C), and the Roland disability questionnaire (D).

Four studies [13], [14], [16], [17] reported visual analogue scale (VAS) scores for low back pain and leg pain. Pooled analysis showed no significant differences between the IS and TDS groups for low back pain (WMD: 1.20; 95% CI: −10.12, 12.53; P = 0.03; I^2^ = 66%) or leg pain (WMD: 7.12; 95% CI: −3.88, 18.12; P = 0.02; I^2^ = 70%; **Fig. 2A, B**). Two studies each reported the results of the ODI [15], [16] and RDQ [15], [17]. Pooled analysis shown no significant difference between the IS and TDS groups for the ODI (WMD: 6.88; 95% CI: −14.92, 28.68; P = 0.03; I^2^ = 79%) or RDQ (WMD: −1.30; 95% CI: −3.07, 0.47; P = 0.88; I^2^ = 0%; **Fig. 1C, D**).

### Complications

**Figure 3:**
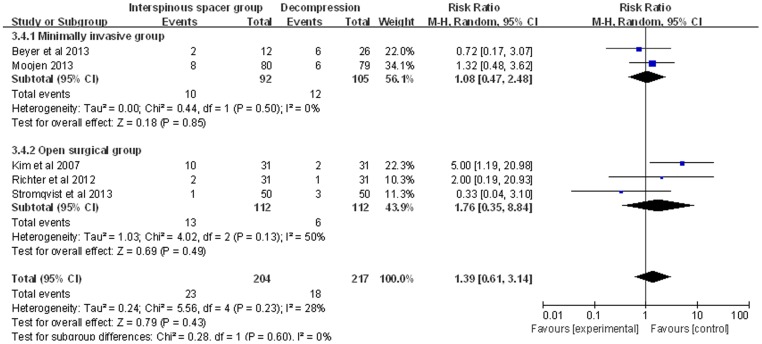
Forest plot showing the meta-analysis for the incidence of complications.

All of the included studies reported the outcome of complications. There were 23/204 complications in the IS group and 18/217 complications in the TDS group. Pooled analysis showed no significant differences between the groups (RR: 1.39; 95% CI: 0.61, 3.14; P = 0.23; I^2^ = 28%; **Fig. 3**). Analyses of the minimally invasive (MI) and open surgery (OS) subgroups revealed no significant differences between the IS and TDS groups (MI subgroup: RR: 1.08; 95% CI: 0.47, 2.48; OS subgroup: RR: 1.76; 95% CI: 0.35, 8.84; **Fig. 3**).

### Reoperation

**Figure 4:**
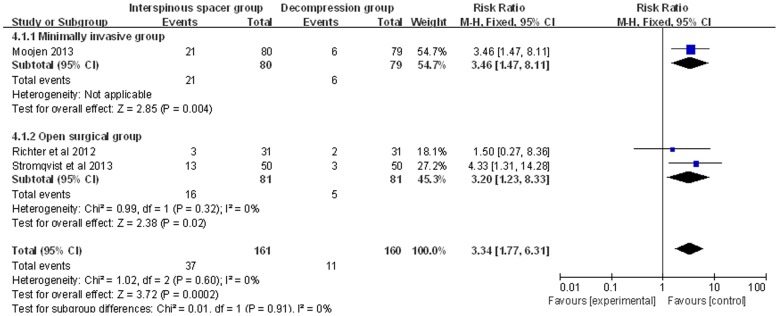
Forest plot showing the meta-analysis for the reoperation rate.

The incidence of reoperation was reported in three studies [13], [15], [17]. In the IS group, 31/161 cases required a second operation, compared to 11/160 cases in the TDS group. The incidence of reoperation was significantly lower in the TDS group (RR: 3.34; 95% CI: 1.77, 6.31; P = 0.60; I^2^ = 0%, **Fig. 4** **)**. Analyses of the MI and OS subgroups showed a higher incidence of reoperation in the IS group (MI subgroup: RR: 3.46; 95% CI: 1.47, 8.11; OS subgroup: RR: 3.20; 95% CI: 1.23, 8.33; **Fig. 4**).

## Discussion

Patients with lumbar spinal stenosis can experience intermittent neurogenic claudication, pain, and numbness in the legs. Implantation of an interspinous spacer can increase the cross-sectional area of the spinal canal [18]. Many interspinous spacers have been designed [19] for clinical use [20], and an increasing number of studies have reported their use for the treatment of degenerative lumbar spinal stenosis [4], [5], [21], [22]. However, most of these studies were case series or clinical experiments without contrasting controls [23]. Our eligibility criteria permitted only five studies to be included in our meta-analysis. Although the included sample size was not large, it is larger than most other studies of dynamic device use for the management of lumbar spinal stenosis [24], [25]. All of the included studies were prospective and comparatively designed, and two were RCTs [13]. Therefore, the results of our meta-analysis are credible.

The meta-analysis revealed no statistically significant differences in the clinical outcomes for back/leg pain, ODI, and RDQ between the two groups. Complication rates were also similar, although the incidence of complications in the TDS group (18/217; 8.3%) was slightly lower than that in the IS group (23/204; 11.3%). Interspinous spacer insertion has been associated with spinous process fracture, implant dislocation, [14], [15], and heterotopic ossification [26], [27], which may explain the slightly higher rate of complications in the IS group.

The reoperation rate was significantly higher in the IS group (37/161; 23.0%) compared to the TDS group (11/160, 6.9%). Moojen et al. [17] and Beyer et al. [16] implanted interspinous spacers through an MI method. Therefore, we performed a subgroup analysis of the incidence rates of complications and reoperations for the MI and OS subgroups. The subgroup analysis revealed the same results as the overall analysis, indicating that the higher reoperation rate was not related to the surgical method (MI or OS). Many surgeons choose indirect decompressive surgery [13] or simply insert the implant percutaneously [28], [29]. It may be that patients could obtain benefits from the MI technique; however, the cost of each interspinous process device is at least €2,000 (£1,704; $2,756), as reported by Moojen et al. [17]. An alternative might be the use of microsurgical technique of simple decompression without interspinous spacer use, many of which have been reported.

A limitation of this meta-analysis was that only five studies were included. There is a lack of studies comparing interspinous spacer use and traditional decompressive surgery in the published literature. Based on the above meta-analysis of 12 to 24 months of clinical results, we conclude that patients may obtain some benefit from MI techniques with interspinous spacer use. However, the high cost and high reoperation rate associated with interspinous spacer use are worrisome. Therefore, the indications, risks, and benefits of using an interspinous process device should be carefully considered before surgery.

## Supporting Information

Table S1 **Data extraction form.**
(DOCX)

Table S2 **Downs and Black Checklist.**
(DOC)

Checklist S1 **PRISMA Checklist.**
(DOC)
