# Supplementary material for: Interspinous Spacer versus Traditional Decompressive Surgery for Lumbar Spinal Stenosis: A Systematic Review and Meta-Analysis
Source: PLoS One. 2014 May 8;9(5):e97142. doi: 10.1371/journal.pone.0097142 (PMC4014612; doi:10.1371/journal.pone.0097142)
Supplement: Table S1 — Data extraction form. (DOCX) [file pone.0097142.s001.docx]

**Data extraction form**

**Source :**

Data extractor:

Date of completing form:

Article title :

Study ID .:

The source of information :

Year of publication of study:

Notes:

**Eligibility**

Confirm eligibility for review:

Reason for exclusion:

**Methods**

Study design:

Randomised:

Sequence generation:

Allocation sequence concealment:

Blinding:

Other concerns about bias:

**Participants**

Total number. IS group: TDS group:

Age:

Sex:

Setting:

Country:

Diagnosis:

**Interventions**

IS group:

TDS group:

**Outcomes**

Follow-up time points

Missing participants

| **Time point** |  |  |  |  |  |  |
| --- | --- | --- | --- | --- | --- | --- |
| **VAS of lower back pain** |  |  |  |  |  |  |
| **VAS of leg pain** |  |  |  |  |  |  |
| **Oswestry disability index** |  |  |  |  |  |  |
| **Roland disability questionnaire** |  |  |  |  |  |  |
| **Others** |  |  |  |  |  |  |
|  |  |  |  |  |  |  |
|  |  |  |  |  |  |  |

**Complications**

|  | Number/total | events |
| --- | --- | --- |
| IS group |  |  |
| TDS group |  |  |

**Reoperation**

|  | Number/total | Reasons, if have |
| --- | --- | --- |
| IS group |  |  |
| TDS group |  |  |
