# Supplementary material for: Interspinous Spacer versus Traditional Decompressive Surgery for Lumbar Spinal Stenosis: A Systematic Review and Meta-Analysis
Source: PLoS One. 2014 May 8;9(5):e97142. doi: 10.1371/journal.pone.0097142 (PMC4014612; doi:10.1371/journal.pone.0097142)
Supplement: Table S2 — Downs and Black Checklist. (DOC) [file pone.0097142.s002.doc]

Table S2: An assessment of risk bias of included studies by Downs and Black checklist.

| Questions of Downs and Black checklist | Stromqvist  2013 | Beyer  2013 | Richter  2012 | Kim  2007 | Moojen  2013 |
| --- | --- | --- | --- | --- | --- |
| Q1: Aim clearly described | Yes | Yes | Yes | Yes | Yes |
| Q2: Outcomes clearly described | Yes | Yes | Yes | Yes | Yes |
| Q3: Characteristics of the patients clearly described | Yes | Yes | Yes | Yes | Yes |
| Q4: Interventions clearly described | Yes | Yes | Yes | Yes | Yes |
| Q5: Principal confounders clearly described | partially | No | No | No | partially |
| Q6: Main findings clearly described | Yes | Yes | Yes | Yes | Yes |
| Q7: Random variability for the main outcomes provided | Yes | Yes | Yes | Yes | Yes |
| Q8: Adverse events reported | Yes | Yes | Yes | Yes | Yes |
| Q9: Lost to follow-up described | Yes | Yes | Yes | Yes | Yes |
| Q10: Actual P-values reported | Yes | No | Yes | No | Yes |
| Q11: Subjects asked to participate representative of the entire population | UD | UD | UD | UD | UD |
| Q12: Subjects prepared to participate representative of the entire population | UD | UD | UD | UD | UD |
| Q13: Staff representative of patient’s environments | UD | UD | UD | UD | UD |
| Q14: Attempt to blind participants | No | No | No | No | Yes |
| Q15: Attempt to blind assessors | No | No | No | No | Yes |
| Q16: Data dredging results stated clearly | Yes | Yes | Yes | Yes | Yes |
| Q17: Analysis adjusted for length of follow up | Yes | Yes | Yes | No | Yes |
| Q18: Appropriate statistics | Yes | Yes | Yes | Yes | Yes |
| Q19: Reliable compliance | UD | UD | UD | UD | UD |
| Q20: Accurate outcome measures | yes | Yes | Yes | Yes | Yes |
| Q21: Same population | Yes | Yes | Yes | Yes | Yes |
| Q22: Participants recruited at the same time | Yes | Yes | Yes | Yes | Yes |
| Q23: Randomised | Yes | NO | No | No | Yes |
| Q24: Adequate allocation concealment | Yes | No | No | No | Yes |
| Q25: Adequate adjustment for confounders | No | No | No | No | No |
| Q26: Loss of follow up reported | Yes | Yes | Yes | Yes | Yes |
| Q27: Power calculation | <n_1_ | <n_1_ | <n_1_ | <n_1_ | <n_1_ |
| **Note:** UD: Unable to determine | | | | |  |
